# The Late Professor Bennett

**Published:** 1831

**Authors:** 


### The late Professor Bennett.

This able teacher has paid the debt of nature since our last number was published; and as no account of his illness has yet been given to the public, we can supply the necessary information, the Editor of this Journal having attended Mr. Bennett during his last and fatal illness, as well as in many preceding attacks of the disease which ultimately terminated this rising young anatomist's existence. There is little doubt that Mr. B. laid the foundation of his ill health in the dissecting-rooms of Paris, where he laboured to improve himself and others, with more zeal than discretion. The consequence was, a very disordered condition of function in all the great abdominal viscera—more especially of the stomach, liver, intestines, and kidneys. The biliary secretion was always going wrong, and presenting unequivocal signs of vitiation. The intestinal secretions and excretions were also particularly deranged, both in colour, smell, and all physical properties. A remarkable symptom, in this respect, was the frequent evacuation of whitish and sometimes fatty or gelatinous fæces, which emitted a strong ammoniacal odour, and were attended with great irritation, both of mind and body. These were the ordinary symptoms; but, in the course of the last eighteen months of his life, he had several attacks of an alarming nature, amounting, upon two occasions at least, to ileus. Obstruction in the bowels, resistance to every kind of aperient, vomiting, distention of the abdomen, pain and tenderness there, ejection of fæcal matters by the mouth, hiccup, facies hippocratica, and small quick pulse, have more than once continued for some days in succession and rendered his case almost hopeless. Nothing but calomel and opium, with large and reiterated injections of soap and water, saved him upon these occasions. But Mr. Bennett had insuperable objections, or rather antipathies to some medicines, especially opium, and often allowed his disease to get to a great height before he would submit to the directions of his medical advisers. In the intervals between these attacks, and during the existence of the ordinary symptoms of derangement of the abdominal functions, before alluded to, Mr. Bennett was extremely imprudent in his diet, drink, and general regimen. He was perpetually changing his plans, and taking all kinds of medicines, except those that were proper for him. The most prudent thing which he did, during the Summer of 1830, was to run away from London, and travel for his health. He came back at the commencement of the Winter lectures, considerably recruited in his bodily functions; but still evincing the germs of a serious disease in the intestinal canal. He resumed his duties, as a teacher in the London University, and, being very imprudent in diet, soon relapsed into a very bad state of health. About a month or six weeks before his death, he had one of his usual attacks of ileus; and his medical attendants almost despaired of effecting a passage through his bowels. The obstruction, however, was at length conquered; but he never afterwards rallied. He was harassed with diarrhœa, and in the motions appeared purulent and other matters, indicating ulceration of some part of the intestinal canal. He daily emaciated — became affected with cough, ulcerated throat, œdema of the lower extremities, loss of voice, and died a mere skeleton! During the last fortnight of his illness Drs. Chambers and Clark joined in consultation with Dr. Johnson, who attended him daily throughout the whole period of his distressing disease. Strange to say, this accomplished and zealous anatomist gave the most positive directions and injunctions that no examination of his body should take place after his decease! These injunctions were obeyed, in order that the feelings of two amiable sisters, who nursed him on his bed of sickness and death, might be spared. As the writer of this short account was only acquainted with Mr. Bennett in the quality of his physician, he feels himself incompetent to give any biographical particulars, which he leaves to some of Mr. Bennett's more intimate friends.

